# Depression and termination of pregnancy (induced abortion) in a national cohort of young Australian women: the confounding effect of women's experience of violence

**DOI:** 10.1186/1471-2458-8-75

**Published:** 2008-02-26

**Authors:** Angela J Taft, Lyndsey F Watson

**Affiliations:** 1 Mother and Child Health Research, La Trobe University, 324-328 Little Lonsdale Street, Melbourne Vic 3000, Australia

## Abstract

**Background:**

Termination of pregnancy is a common and safe medical procedure in countries where it is legal. One in four Australian women terminates a pregnancy, most often when young. There is inconclusive evidence about whether pregnancy termination affects women's rates of depression. There is evidence of a strong association between partner violence and depression.

Our objective was to examine the associations with depression of women's experience of violence, pregnancy termination, births and socio-demographic characteristics, among a population-based sample of young Australian women.

**Methods:**

The data from the Younger cohort of the Australian Longitudinal Study on Women's Health comprised 14,776 women aged 18–23 in Survey I (1996) of whom 9683 aged 22–27 also responded to Survey 2 (2000). With linked data, we distinguished terminations, violence and depression reported before and after 1996.

We used logistic regression to examine the association of depression (CES-D 10) as both a dichotomous and linear measure in 2000 with pregnancy termination, numbers of births and with violence separately and then in mutually adjusted models with sociodemographic variables.

**Results:**

30% of young women were depressed. Eleven percent (n = 1076) reported a termination by 2000. A first termination before 1996 and between 1996 and 2000 were both associated with depression in a univariate model (OR 1.37, 95%CI 1.12 to 1.66; OR 1.52, 95%CI 1.24 to 1.87). However, after adjustment for violence, numbers of births and sociodemographic variables (OR 1.22, 95%CI 0.99 to 1.51) this became only marginally significant, a similar association with having two or more births (1.26, 95%CI. 1.00 to 1.58).

In contrast, any form of violence but especially that of partner violence in 1996 or 2000, was significantly associated with depression: in univariate (OR 2.31, 95%CI 1.97 to 2.70 or 2.45, 95% CI 1.99 to 3.04) and multivariate models (AOR 2.06, 95%CI 1.74 to 2.43 or 2.12, 95%CI 1.69 to 2.65). Linear regression showed a four fold greater effect of violence than termination or births.

**Conclusion:**

Violence, especially partner violence, makes a significantly greater contribution to women's depression compared with pregnancy termination or births. Any strategy to reduce the burden of women's depression should include prevention or reduction of violence against women and strengthening women's sexual and reproductive health to ensure that pregnancies are planned and wanted.

## Background

Termination of pregnancy (also referred to as induced abortion or more commonly just abortion) is one of the most common and safe medical procedures in Australia, as it is in other developed countries worldwide. In Australia, around one in four women terminate pregnancies, most often in their early twenties [1]. Termination is most frequently a woman's response to unwanted pregnancy, due to a range of possible circumstances e.g. poor access to contraception, contraceptive failure, unprotected intercourse or coerced sex occurring in the often complex context of her life situation [2]. The question of whether to terminate a pregnancy often forces women to confront their current and potential life circumstances, including that of their intimate relationships, employment, financial situation and readiness to parent. Our previous analyses of data from this cohort of young Australian women found that young women experiencing partner violence were more socially disadvantaged, were pregnant more often than non-abused women and were more likely to terminate their pregnancies than those better off and violence-free. A higher proportion of younger women (particularly those in their teenage years) reported termination of pregnancies (occurring before the 1996 survey), compared with the proportion reporting this in the 2000 survey, when aged in their later twenties [3,4].

Termination of pregnancy remains a controversial issue, with some arguing that it leads to adverse mental health sequelae [5,6] and others that the evidence suggests the contrary [7,8]. There are major limitations with the evidence to date [6]: significant under-reporting, lack of clarity about whether the probable depression or termination came first; limited sample sizes or study design, clinical rather than population or community samples; and confounding factors for which no adjustment has been made. Important potential confounding factors contributing to depression, such as partner violence, childhood sexual abuse or socioeconomic disadvantage may not be taken into account. There is now consistent and reliable evidence of the association between partner violence and depression [9,10] and between partner violence and termination of pregnancy [3,11,12].

The longitudinal population study by Fergusson et al of young New Zealand women between the ages of 15–25 did not include a measure of partner violence either in the family of origin or among the young people themselves [6], but did include some measures of problematic family functioning such as changes of parents, parental history of criminality and experience of childhood sexual abuse. The authors suggested that abortion/termination among teenage women and women in their early twenties may be associated with an increased likelihood of mental health problems.

An early analysis of data from the US national longitudinal survey of young women aged 14–21 in 1979 had concluded that pregnancy termination placed young people at risk of depression [5]. Schmiege and Russo reanalysed these data, arguing that the previous analysis had misclassified subjects (unintended rather than unwanted pregnancies) and excluded women (who subsequently terminated following an index pregnancy) who should have been included. They controlled for the same social and personal factors as the first analysis, but found that among those young women with an unwanted pregnancy, there was no difference in levels of depression between those who terminated the pregnancy, compared with those who continued the unwanted pregnancy to term [8].

We undertook a linked analysis of the 1996 and 2000 surveys of the Younger cohort of the Australian Longitudinal Study of Women's Health (ALSWH) to examine associations of probable current depression with termination of pregnancy and women's experiences of violence among a random population sample of young Australian women.

## Methods

### The data

The ALSWH has been described in detail elsewhere [13]. Briefly, the project involves three age cohorts of women, who were 18–23 years (the Younger cohort), 45–50 years (the Mid-age cohort) and 70–75 years (the Older cohort) when first surveyed in 1996. They are being followed longitudinally for at least twenty years. Participants respond to mailed surveys covering aspects of physical and emotional health, health service use, demographics, time use, health behaviours, life events, and other variables to develop a comprehensive picture of women's health in its social context. About 36,000 women aged 18–23, selected from the Australian national health insurance database were eligible for the Younger cohort. 14,779 women responded to Survey 1 in 1996. Data for 9683 who responded to Survey 2 in 2000 were linked to their responses from the Survey 1 in 1996 for this analysis.

The project as a whole has ethics clearance from the Human Research Ethics Committees at the Universities of Newcastle and Queensland, Australia. The Human Research Ethics Committee of La Trobe University (Reference 01/140), Melbourne, Australia, gave approval for the secondary data analysis in this study.

Sample selection involved over-sampling in rural and remote areas; therefore, in the analysis, we incorporated probability weights to reflect the population distribution of Australian women in the age group studied [13]. This weighting resulted in an estimated sample size of 9,692.

### Measures

#### Measure of probable depression: the CES-D 10

The Centre for Epidemiological Studies Depression Scale (CES-D) is a 20-item self-report scale used to identify the persistence and severity of probable depression over the past week among the general population. It was constructed from well-established existing scales and has been reduced in length by half (10-item CES-D 10) without significant loss in specificity or sensitivity. It is not a diagnostic tool but higher scores indicate probable depression (high levels of depressive symptomatology). The ALSWH incorporates the 10 item CES-D, where ≥ 10 is regarded as an appropriate cutoff and this is used in the current analysis [14].

The ALSWH also included a question in 2000 about depression – 'has a doctor ever told you that you have depression?' This question was asked of the past four years (1996–2000) and over four years ago. The answer to this question over four years ago was used to control for depression prior to 1996.

#### Socio-demographic factors

We included the following socio-demographic variables of interest in the model: marital status, age, highest education level, occupation, private health insurance cover, country of birth, area and state of residence (as the legal status and accessibility of pregnancy termination services varies by state). Whilst indigenous (Aboriginal and Torres Strait Islander – ATSI) status was included in the analyses, associations are not reported in accordance with ALSWH requirements to ensure results cannot be misinterpreted as the ATSI sample is not representative.

#### Reproductive events

The survey questionnaires for Surveys 1 and 2 asked women to list how many times they had (a) been pregnant, (b) had a miscarriage, (c) had a pregnancy termination and (d) given birth at term or (e) given birth at less than 37 weeks – preterm. For this paper, the responses to (c) were dichotomized into 'ever' and 'never'; multiple events were rarely reported and the power for detecting significant associations for these was low. We linked the termination data from women's responses in the two surveys, and were thus able to distinguish those who reported their first termination prior to the 1996 survey when they were either teenage or in their early twenties from those who first reported a termination in 2000. Responses to (d) and (e) were also combined to give a variable for term and preterm births. This variable was stratified into no births, one and two or more births.

#### Composite variable for violence

Intimate partner violence, often called domestic violence, is a complex, multi-dimensional set of coercive behaviours, most often measured by acts of physical and sexual violence. However, women who are physically abused by intimate partners are often abused in other ways, for instance psychologically or economically. Three short questions about violence were asked in the ALSWH in 1996 and 2000:

• In the last twelve months, have you been pushed, grabbed, shoved, kicked or hit?

• In the last twelve months, have you been forced to take part in unwanted sexual activity?

• Have you ever been in a violent relationship with a partner/spouse?

The first questions were taken from the Conflict Tactics Scale [15] to enable comparisons with those used in the Australian population study being undertaken by the Australian Bureau of Statistics at the same time [16]. The final question aimed to enable distinction between women who reported partner violence and those who did not. We used combined responses to distinguish women reporting partner violence and those reporting recent physical or sexual abuse perpetrated by other family members or acquaintances. We produced a composite variable with five mutually exclusive categories determined, after preliminary cross-tabulations, to indicate the reported experience of violence at survey I (1996) and for the first time at Survey 2 in 2000. A small group with missing data on these factors formed a sixth group. The categories were also of sufficient size to provide reliable estimates.

1. No violence (NoV)

2. Partner violence – reported in 1996 or both in 1996 and 2000 (PV 1996)

3. Partner violence – reported in 2000 but not in 1996 (PV 2000)

4. Recent sexual or physical violence but no partner violence in 1996 and/or 2000 (OV 1996)

5. Recent sexual or physical violence but no partner violence first in 2000 (OV 2000)

6. Missing responses on any of the questions

### Analyses

Using the data for all women from Survey 2 in 2000, we estimated the associations between probable depression and sociodemographic factors (Table 1).

**Table 1:** Probable depression and associations with sociodemographic factors. Survey 2 of the Younger cohort of the Australian Longitudinal Study on Women's Health 2000

			Depressed (presumed from CESD ≥ 10)		
			
Variable	Total^1^	*Column%*	%	AOR^2 3^	95% CI
Total	9,327	*100*	*30*		
Marital status					
Single	5,193	*56*	*33*	1.00	
Married	2,114	*23*	*25*	0.61 ***	0.54 – 0.70
De facto	1,798	*19*	*28*	0.76 ***	0.67 – 0.87
Wid/sep/divorced	183	*2*	*40*	1.13	0.81 – 1.58
Age (at response to Survey 2)					
<24 years	2,326	*25*	*32*	1.00	
≥ 24 years	7,007	*75*	*30*	0.99	0.88 – 1.11
Education level					
None/year10 education	821	*9*	*38*	1.07	0.89 – 1.28
HSC	2,027	*23*	*35*	1.00	
App/cert	2,085	*23*	*30*	0.83 *	0.72 – 0.96
Degree or higher	4,098	*45*	*26*	0.72 ***	0.62 – 0.83
Occupation					
Manager/professional	3,960	*44*	*26*	1.00	
Para-professional	593	*7*	*26*	0.93	0.75 – 1.16
Trade/clerical	2,721	*31*	*32*	1.23 **	1.07 – 1.41
Blue-collar	514	*6*	*32*	1.17	0.93 – 1.47
No paid job	261	*3*	*33*	1.18	0.88 – 1.58
Other^4^	773	*9*	*39*	1.76 ***	1.45 – 2.14
Health insurance					
No private cover	6,228	*67*	*32*	1.00	
Has private cover	3,043	*33*	*27*	0.85 **	0.76 – 0.95
Country of birth					
Australian born	8,838	*95*	*30*	1.00	
European born	107	*1*	*30*	0.96	0.58 – 1.57
Asian born	323	*4*	*37*	1.32 *	1.00 – 1.74
Area of residence					
Urban	6,682	*75*	*31*	1.00	
Rural	1,989	*22*	*28*	0.79 ***	0.71 – 0.88
Remote	302	*3*	*30*	0.92	0.72 – 1.18

1 Total numbers within variables vary due to missing responses, column %'s calculates without including missing category

2 Adjusted odds ratio, mutually adjusted for all other sociodemographic factors in table as well as Aboriginal and Torres Strait identity.

3 ^M ^p < 0.10, * p ≤ 0.05, ** p ≤ 0.01, *** p ≤ 0.001

4 Other includes home duties, group comprises women more likely to be married and to have children than those in other groups

The associations between depression, termination, births and violence were then estimated first using univariate and then multivariate logistic regression. Using univariable logistic regression, we estimated associations with probable depression in 2000 between women first reporting terminations in the 1996 survey when they were aged between 18 and 23 or in the 2000 survey (mutually exclusive) when they were aged between 22 and 27, compared with those never reporting termination at either age. We also estimated the association with women reporting no births or one or more births and for any form of violence women experienced prior to 1996 and between 1996 and 2000.

We modeled the effects on depression in 2000 as a dichotomous (with a score of less than 10 or 10 or more on the CESD) and linear variable, controlling for prior depression (depression in 1996 as reported from a doctor) adjusting for violence in each time period, the numbers of births women reported and socio-demographic factors (Table 2).

**Table 2:** Association of violence and termination with CESD. Survey 2 of the Younger cohort of the Australian Longitudinal Study of Women's Health 2000

			Univariate		Multivariate & SES	
				
Variable	*Column% *(n = 9333)	% Depressed	OR	95% CI	AOR^2, 1^	95% CI
Termination						
No termn	*89*	*29*	1.00		1.00	
Termn 1st in 1996	*6*	*36*	1.37 ***	1.12 – 1.66	1.04	0.85 – 1.29
Termn 1st in 2000	*5*	*38*	1.52 ***	1.24 – 1.87	1.22 ^M^	0.99 – 1.51
Number of births						
0	*84*	*29*	1.00		1.00	
1	*10*	*33*	1.20 *	1.03 – 1.40	1.04	0.87 – 1.25
2 or more	*6*	*37*	1.44 ***	1.21 – 1.72	1.26 *	1.00 – 1.58
Violence						
NonV	*66*	*25*	1.00		1.00	
PV 1996	*9*	*43*	2.31 ***	1.97 – 2.70	2.06 ***	1.74 – 2.43
PV 2000	*5*	*45*	2.45 ***	1.99 – 3.04	2.12 ***	1.69 – 2.65
OV 1996	*12*	*38*	1.83 ***	1.58 – 2.13	1.77 ***	1.51 – 2.06
OV 2000	*8*	*41*	2.10 ***	1.77 – 2.49	2.00 ***	1.68 – 2.38
Depression in 1996						
No	*96*	*29*	1.00		1.00	
Yes	*4*	*42*	1.81 ***	1.38 – 2.39	1.57 **	1.17 – 2.10

1 ^M ^p < 0.10, * p ≤ 0.05, ** p ≤ 0.01, *** p ≤ 0.001

2 Mutually adjusted for termination violence, depression in 1996 and number of children as well as adjusted for marital status, age, education level, occupation, health insurance status, country of birth, area of residence, state of residence and Aboriginal or Torres Strait identity.

In all cases, numbers (n) and percentages reported have been adjusted for the area weighting. All reported odds ratios (ORs) or adjusted odds ratios (AORs), 95% confidence intervals (CIs), and p-value indicators are adjusted for population sample area weighting. All analyses were using survey commands in Stata 8. [17]

Missing data (less than 3% for any variable) were accounted for by an indicator variable in the models and have not been reported here.

## Results

### Young women, probable depression and socio-demographic factors (Table 1)

We found that 30% of these young women aged between 22–27 years scored 10 or above on the CESD scale indicating probable depression, when surveyed in 2000. Women who were married or living in de facto relationships were less likely to report depression (OR 0.61, 95% CI 0.54 to 0.70; OR 0.76, 95% CI 0.67 to 0.87 respectively) than single or divorced/separated women. Women with post-secondary qualifications, especially tertiary degrees, those with private health insurance and those living in rural areas were also less likely to be depressed compared with women with secondary education only, no private health insurance and those living in urban areas respectively. Woman with clerical or trade jobs or those with home duties were more likely to be depressed (OR 1.23, 95% CI 1.07 to 1.41; OR 1.76, 95% CI 1.45 to 2.14) than managerial or professional women.

Having a CESD score of greater than 10 was significantly associated with being diagnosed with depression by a doctor. Twenty-five percent of women having never been diagnosed with depression scored greater than 10 on the CESD compared with 39% for those diagnosed before 1996 and 56% for those diagnosed since 1996 (OR 1.94, 95%CI 1.43 to 2.63; OR 3.84, 95% CI 3.32 to 4.42 respectively).

### The associations between depression, termination of pregnancy, numbers of births and forms of violence against young women (Table 2)

At the time of the 1996 survey (when women were aged between 18 and 23), only 12% of young women had ever been pregnant (data not shown). Six percent (n = 559) reported a termination (50% of those ever pregnant). At the time of the 2000 survey, 30% of young women aged between 22 and 27 had ever been pregnant. Eleven percent (n = 1076) reported terminating their pregnancies, of whom five percent reported a termination for the first time in the 2000 survey. Overall, 37% of those who reported being pregnant since 1996 had terminated a pregnancy.

Among women reporting no violence, 25% scored as depressed. In contrast 43% of those women reporting partner violence in 1996 scored as depressed and 45% reporting partner violence in 2000. Thirty-eight percent of women in 1996 and 41% in 2000 reporting 'other violence' reported depression.

While 29% of women having had no births scored as depressed, 33% of those with one birth and 37% of those with two or more scored as depressed.

In univariate analyses of the relationship between termination and probable depression (Table 2), there is a significant association, both with a termination reported prior to 1996 (OR 1.37, 95%CI 1.12 to 1.66) or termination between 1996 and 2000 (OR 1.52, 95%CI 1.24 to 1.87). There is a similar association with having two or more births (OR 1.44, 95%CI 1.21 to 1.72).

Further adjustment for socio-demographic factors reduces the odds ratios associated with pregnancy termination, whilst those associated with women's experiences of violence remain high and statistically significant. The association is no longer statistically significant for a termination reported before 1996 (AOR 1.04, 95%CI 0.85 to 1.29), or for those reported later (AOR 1.22, 95% CI 0.99 to 1.51). A very similar pattern emerges in an adjusted model for having births. The effect size vanishes for one birth (OR = 1.04, 95% CI 0.87 to 1.25) while the effect of two or more births drops (OR = 1.26 95% CI 1.00 to 1.58).

In contrast, the effect of violence on depression remains significant. Compared with women experiencing no violence, the odds ratios for scoring as depressed are highest for women who report experiencing partner violence in 2000 (AOR 2.12, 95%CI 1.69 to 2.65) followed by partner violence in 1996 (AOR 2.06, 95%CI 1.74 to 2.43). Women reporting other violence in 1996 and 2000 have a reduced but still significant odds ratio for probable depression (AOR 1.77, 95%CI 1.51 to 2.06; 2.00, 95%CI 1.68 to 2.38).

An interaction between violence and pregnancy termination in their association with probable depression was not able to be demonstrated since the odds ratios for pregnancy terminations remained the same for each level of violence experienced (data not shown).

The magnitude of effect differences is very clear when considering the relative contributions each factor makes to an additional unit increase on the CESD depression scale when estimated using linear regression. The overall mean for CESD was 7.60, with a standard error of 0.06 and a range from 0 to 30. The non-significant coefficients for first abortion in 1996 and 2000 are 0.31 (95%CI -0.21 to 0.83) and 0.51 (95%CI -0.02 to 1.04) respectively and for one birth and 2+ births 0.04 (95% CI -0.39 to 0.47) and 0.29 (95%CI -0.26 to 0.84) respectively. In contrast, the significant effects of violence on depression are four times as large – partner violence in 1996 contributes an additional 2.28 (95%CI 1.82 to 2.74) and in 2000, 2.24 (95%CI 1.58 to 2.90) to each unit on the depression scale. Violence from others in 1996 and 2000 contributes 1.60 (95%CI 1.20 to 2.00) and 2.12 (95%CI 1.62 to 2.62) respectively.

## Discussion

We found, similar to France et al [14], that 30% of young women scored as probably depressed. Poverty and other socio-demographic disadvantage has previously been shown to impact significantly on women's experience of depression [18]. We have previously published data from this cohort of women showing evidence of an association between socio-economic disadvantage and pregnancy terminations [3].

More recently, women's experiences of violence, especially partner violence, have been convincingly demonstrated to have an association with depression [9,10]. Our previous analyses of these data demonstrated that partner violence has a strong association with pregnancy termination; also found elsewhere [3,12]. In addition, we found that when women report abuse by partners, they experience greater likelihood of more frequent pregnancies, miscarriages and adverse pregnancy outcomes and this troubled reproductive history should be considered in relation to maternal depression.

Some authors have argued that pregnancy termination or induced abortion plays an aetiological role in depression [19], therefore our current analysis is timely. This study adds to the accumulation of evidence that termination has a borderline association with depression, which is an equivalent strength of association with that of having two or more births. Whilst there appears to be a slight effect, it is reduced when other more powerful influences especially that of partner violence are taken into account and may have occurred by chance. The consistency of associations in our analyses emphasises that pregnancy termination or having two or more births at an early age is less likely to be associated with depression compared with that of partner or other violence. [7]. What this study suggests, as others have previously [7,9,10], is that women's experience of violence – likely to continue over an extended period of time – along with other adverse reproductive events and circumstances increases their risk of depression, rather than their experience of terminating a pregnancy. Our findings suggest that studies which find an association between termination and depression or suggest a link between termination and suicide, but have failed to consider partner violence as a confounder are potentially flawed [5,19,20].

The study has strength as it is based on a large longitudinal national randomly selected population sample (including over 1000 women who reported terminations) with linked data. Our finding of the lack of interaction between violence and pregnancy termination in their association with depression suggests that the link between mental health and exposure to termination of pregnancy found by Fergusson et al in their longitudinal sample might be the result of failing to take current or previous experience of partner violence into account, as they indeed surmised [6].

The study has limitations. ALSWH is limited by a relatively low response rate and similar to many mailed surveys, tends to a higher socio-demographic group of women. This response bias, the young age of the women (22–27) and the fact that abused women were less likely to respond at survey 2 in 2000 goes some way to help explain the under-reporting of abortion in this survey, compared with the overall community. This is likely to contribute to under-estimates of terminations, compared with the national estimates [1].

The questions in the ALSWH about a violent relationship with a partner have not been validated and may be open to misinterpretation. Nevertheless, associations with our composite variable have been consistent with other studies [11,16,21,22]. In addition, the question about depression diagnosed by a doctor will be open to recall bias. However, there was a significant association between this and having a CESD score of greater than 10. Depression diagnosed before 1996 was controlled for in our models. On the other hand, this measure was different from the CESD and refers to all previous points in respondents' lives. With a more specific control for pre-existing depression in 1996, the association between termination and depression may have been further reduced.

In intimate relationships, women and their partners have many issues to consider when faced with an unwanted or unexpected pregnancy. These include making judgments about financial security, employment prospects, maturity and readiness to parent. If partner violence is present in the relationship, it seems plausible that the cumulative impact of the abuse, perhaps several pregnancies, adverse pregnancy outcomes, numbers of children and poverty will have a greater impact on a women's mental health than any decision to terminate a pregnancy. The strong and consistent finding of a link between partner violence and depression and the relationship between violence and pregnancy termination helps to explain the growing evidence of the lack of significant association between depression and pregnancy termination.

## Conclusion

The evidence in this paper casts doubt on the thesis that there is a significant causal link between termination and depression; instead it confirms that there is a strong significant link between depression and women's experience of partner violence. Although the association found between depression and prior termination is not statistically significant, its size and near significance might lead one to see it as evidence that choosing pregnancy termination over childbirth increases the risk of depression in younger women. We believe this would be a mistake in view of the study's findings. For women deciding how to resolve an unwanted pregnancy, the study demonstrates the likelihood that continuing an unintended pregnancy would result in as much a risk of depression as terminating the pregnancy. In fact, our finding of depression among young women with two or more children suggests that an unwanted birth might slightly risk an adverse effect on a young woman's mental health.

Termination is most commonly the result of an unplanned and unwanted pregnancy. Prevention of unwanted pregnancies is likely to be reduced by a comprehensive sexual and reproductive health program, but also includes tackling structural determinants such as poverty and the prevention of intimate partner violence. Depression contributes to a considerable burden of disease and disability among women worldwide. To reduce the mental ill health associated with reproductive events, national sexual and reproductive health strategies should also incorporate initiatives to reduce violence against women.

## Competing interests

The author(s) declare that they have no competing interests.

## Authors' contributions

Both AT and LW designed the study. LW undertook the statistical analyses and AT wrote the first draft of the article. Both authors revised the manuscript.

## Pre-publication history

The pre-publication history for this paper can be accessed here:


